# Role of gut microbiota and trimethylamine *N*-oxide in preeclampsia: pathophysiological insights and therapeutic opportunities

**DOI:** 10.1097/MS9.0000000000003138

**Published:** 2025-03-27

**Authors:** Manahil Mubeen, Alizah Shazad, Maryam Aziz, Sijan Poudel

**Affiliations:** a Department of Medicine, Dow Medical College, Karachi, Pakistan; b Department of Medicine, Lumbini Medical College & Teaching Hospital, Tansen, Nepal

**Keywords:** endothelial dysfunction, gut microbiota, preeclampsia, pregnancy complications, therapeutic strategies, trimethylamine *N*-oxide (TMAO)

## Abstract

Preeclampsia (PE) is a condition where a sudden rise in blood pressure (>140/90 mmHg) after the 20th week of gestation poses a significant threat to maternal as well as fetal health. PE causes maternal organ damage and several fetal abnormalities which may prove fatal, leading to a high mortality rate. Several studies have been conducted on the role of the gut microbiome, linking its connection to the development of various diseases including PE. One such derivative of gut microbiota is trimethylamine *N*-oxide (TMAO), a metabolite shown to be involved in the progression of PE by disrupting the normal microbiome, promoting inflammation and oxidative stress. The objective of this editorial is to provide a general overview of PE, assess the negative effect of TMAO leading to diseases such as PE, and provide an outline for its potential exploitation as a treatment strategy. Although the current findings offer important insights into the mechanisms related to TMAO, further trials can confirm its clinical relevance and investigate its potential as a diagnostic biomarker and a therapeutic target.

Preeclampsia (PE) is defined as the de novo appearance of hypertension (>140/90mm Hg) after the 20th week of gestation, along with evidence of maternal organ failure, fetal growth restriction, and stillbirth if it is overlooked^[1]^. It is worth noting that more than 50,000 maternal and more than half a million fetal lives are lost due to PE every year^[2]^. PE is characterized by new-onset proteinuria of >300 mg/day or other indications of renal insufficiency, hematological complications such as thrombocytopenia and liver dysfunction, or neurological complications such as visual disturbance and/or evidence of uteroplacental dysfunction, necessitating urgent delivery^[1,2]^. It causes maternal complications such as cerebrovascular bleeding, retinal detachment, HELLP syndrome (**H**emolysis, **E**levated **L**iver enzymes, **L**ow **P**latelet count Syndrome), eclampsia, increased risk of chronic hypertension, ischemic heart disease, and stroke^[1]^. PE puts viable fetuses at risk of restricted growth, perinatal mortality, congenital defects, and premature birth, with increased susceptibility to respiratory disorders. Growing up, such children may also face severe neurological deficits throughout their lives^[2,3]^. Advanced maternal age (over 35), history of PE, obesity (BMI >30), diabetes, chronic hypertension, chronic kidney disease, systemic lupus erythematosus, antiphospholipid syndrome, nulliparity, multifetal pregnancies, and the use of assisted reproductive technologies are amongst the many risk factors associated with this disease^[2,3]^.

Currently, there has been a heightened interest of the health community towards the gut microbiome and its metabolites like trimethylamine *N*-oxide (TMAO), regarding maternal and fetal health^[4,5]^, as well as for several diseases. Gut microbiome, or gut microbiota is defined as a complex assembly of trillions of microorganisms formed in the gastrointestinal tract, which have been proven to play a crucial role in regulating various important body processes. Several recent studies have highlighted that an imbalance in this gut flora leads to PE, warranting further discussion of its role and mechanism^[4,5]^. Our editorial in particular discusses an intestinal microbiota-derived metabolite TMAO, its involvement in PE, and its recognition as a novel treatment strategy, while providing a general overview of the pathogenesis of PE, and the PE-microbiota link.

Pathogenesis of PE includes vascular factors, imbalance of immune balance, systemic inflammatory response, regulation of active peptides, short-chain fatty acids (SCFAs), TMAO, and other metabolites caused by gut flora imbalance^[6]^. During pregnancy, the placenta is important in providing optimal perfusion to the growing fetus. However, abnormal placental development in some patients causes endothelial dysfunction and severe vasoconstriction, contributing to gestational hypertension^[3]^. Vascular factors supporting gestational hypertension include failure of extravillous trophoblast invasion and incomplete spiral artery remodeling^[2]^, leading to high vascular resistance and impaired oxygen delivery to the placenta, which exacerbates placental abnormalities^[2,4]^. These changes further increase the formation of angiogenic markers such as soluble fms-like tyrosine kinase-1 (sFlt-1) and soluble endoglin (sEng), both of which contribute to endothelial dysfunction seen in PE^[2,3]^. Another factor leading to this disruptive pathology is the imbalance of immune responses affecting maternal-fetal tolerance, leading to a heightened inflammatory response^[3,5]^. This promotes oxidative stress and dysregulation of active peptides such as angiotensin II and endothelin-1, intensifying vasoconstriction^[2]^, which reduces blood flow and compromises perfusion to vital organs including placental tissue, causing increased tissue destruction and ischemia that manifests as proteinuria, hepatic, neurological, pulmonary, and renal dysfunction, causing PE^[3]^.

Present management of PE only allows for delivery as the definite treatment in managing the condition, with aspirin and calcium for prophylaxis, but aspirin has very minimal benefit to PE if it is started after 16 weeks of gestation^[2]^. Moreover, a recent meta-analysis highlighted that no systematic studies currently have determined the optimal aspirin dosage for high-risk pregnant women with PE, creating confusion for both healthcare providers and patients^[7]^. This uncertainty is further compounded by the increased risk of side effects, such as gastrointestinal bleeding and potential impacts on the fetal cardiovascular system^[7]^. Antihypertensive drugs for treatment can have side effects, and challenges in making a diagnosis with a lack of biomarkers make management and early intervention for PE even more difficult, raising concerns^[2]^. However, as introduced above, TMAO, a metabolite derived from the gut microbiota, is among the emerging therapeutic targets for PE^[5]^, demanding a deeper understanding of the microbiota-PE link.

During pregnancy, there is a difference in the composition of gut microbiota from the first (T1) to the third (T3) trimester. The abundance of Proteobacteria and Actinobacteria increases from T1 to T3^[8]^. Similarly, T1 microbial composition has greater (α) phylogenetic diversity and taxonomic distributions than T3 samples. The T1 Microbial diversity is normal while the T3 diversity is aberrant because T3 β-diversity is far higher than for T1^[8]^. Normally, this gut microbiota maintains a robust immune function, supporting anti-inflammatory responses, helping manage inflammation, promoting endothelial function, and ensuring metabolic integrity during pregnancy^[9]^. For example, Lipopolysaccharides and SCFAs produced by intestinal bacteria can regulate macrophage activation and inflammation^[10]^, protect placental integrity, and enhance its vascularization^[11]^.

However, an imbalance in the composition and number of these microbes can result in adverse outcomes such as PE. Researchers established PE results from an imbalanced gut microbiome state, with affected women presenting with reduced intestinal diversity combined, harmful bacterial growth, and distinct bacterial profiles when compared to their normal pregnant counterparts^[5,6]^. In particular, a depletion in beneficial bacteria like *Akkermansia muciniphila* that helps to promote host immune homeostasis and maintain the integrity of the gut barrier^[12]^, and *Bifidobacterium*, that plays a protective role against PE and pathogen accumulation by lowering the pH of intestine; results in an enhanced pro-inflammatory state and metabolic dysfunction^[13]^. This dysbiosis disturbs normal gut permeability, allowing toxic metabolites and pro-inflammatory substances to leak into circulation, leading to endothelial dysfunction and subsequent activation of the coagulation cascade, as seen in PE^[13]^.

A healthy microbiome is also required for the complete metabolism of nutrients and to prevent the accumulation of harmful metabolites. If the microbiome is disturbed, it may lead to the accumulation of these unwanted metabolites leading to a flare-up of an inflammatory response and hyperpermeability of intestinal barrier, altering normal physiology and hence leading to complications in pregnancy^[14]^. Among the various metabolites produced, TMAO stands out as a key player. TMAO is produced when dietary choline and carnitine are metabolized by gut microbiota into trimethylamine (TMA), which is then converted to TMAO in the liver by flavin-containing monooxygenases^[6]^. In a healthy pregnancy, gut microbiota facilitates efficient TMAO metabolism and prevents its buildup. However, when there is a microbiota imbalance, such as an overgrowth of TMAO-producing bacteria like *Klebsiella* and *Gemmobacter*, as found in higher abundance in preeclamptic patients, elevated TMAO levels occur^[5]^.

Medical research demonstrated a substantial rise in TMAO levels during pregnancy between the second trimester and delivery, reaching median concentrations of 84.80 μg/m^3^ in the second trimester and 138.60 μg/m^3^ at childbirth point^[15]^. Elevated TMAO levels particularly at the time of delivery were significantly associated with PE^[15]^. Another study conducted at Peking University Third Hospital on a total of 96 singleton pregnancy PE patients also found alterations in gut microbiota composition, increased fecal and plasma LPS levels, and significantly high TMAO concentrations in the PE group as compared to the control group^[16]^.

TMAO play a crucial role in exacerbating the factors involved in pathogenesis of PE. TMAO promotes oxidative stress and inflammation through the activation of NF-kB pathway, increasing the expression of inflammatory cytokines like IL-1B and IL-18^[6,17]^. This creates a vicious cycle of endothelial dysfunction, which impairs the ability of blood vessels to relax, exacerbating hypertension seen in PE women. TMAO also plays a direct role in the pathology of PE by inhibiting nitric oxide (NO) production in endothelial cells, particularly those in the umbilical vein^[6,16]^. NO is critical for vascular relaxation and blood flow regulation, and its suppression leads to vasoconstriction and placental ischemia. In addition, TMAO influences lipid metabolism at multiple anatomic sites, and up-regulation of macrophage scavenger receptors, contributing to the development of atherosclerotic-like conditions in the placenta^[6,16]^. This process leads to lipid accumulation in spiral arteries, defective remodeling, and increased reactive oxygen species in the placenta, all of which are key contributors to exacerbating PE^[5]^.

Furthermore, animal studies conducted by Wang *et al* have demonstrated that targeting TMAO reduction through inhibitors like 3,3-dimethyl-1-butanol (DMB) can improve pregnancy outcomes in PE models^[5]^. In these studies, blocking TMAO led to reduced blood pressure, lower urinary protein levels, and better fetal growth^[5]^. These findings reinforce the idea that elevated TMAO levels are not just a marker but an active participant in the progression of PE, highlighting the potential of targeting TMAO as a therapeutic strategy for managing it.

Numerous strategies can be implemented to maintain TMAO levels within safe and acceptable ranges. As TMAO is derived from compounds like choline and carnitine, both abundant in red meat, reducing its consumption and replacing it with a vegetarian diet is an effective strategy for reducing plasma TMAO levels^[18]^. However, some studies have found that nutrients containing TMA are essential for survival and have a protective effect on the heart^[18]^. Future research should prioritize finding a balance between the cardiovascular advantages of nutrients that contain TMA and the potential risks associated with TMAO formation. Nonetheless, embracing a healthy routine, coupled with dietary modifications helps keep TMAO levels under control. Additionally, medications such as metformin and resveratrol can be used as adjunctive pharmacological supplements, efficiently alleviating cardiovascular complications associated with PE^[18]^.

Various enzymatic pathways have been discovered that help regulate TMAO levels. One such enzyme is flavin-containing monooxygenase, which helps convert TMA to TMAO. According to research, inhibiting the enzyme helps reduce TMAO levels, hence producing encouraging results^[17]^. DMB, is known to decrease the amount of TMAO precursors. It inhibits the enzyme choline TMA lyase that cleaves choline to produce TMA to be used for TMAO production, but it is unable to completely prevent TMAO formation and does not inhibit the formation of TMA from γ-butyrobetaine^[17]^. Meldonium however, which is a carnitine analogue recognized for its anti-atherosclerotic and anti-ischemic effects, can inhibit the formation of bacterial TMAO. It achieves this by competitively inhibiting bacterial carnitine palmitoyl transferase-1 (CPT-1), thereby reducing the production of TMA. Furthermore, prebiotics and probiotics are well-known to help repopulate the gut with healthy microbes, thus aiding in reducing TMAO levels^[17]^. However, along with numerous benefits, targeting TMAO levels using these strategies poses challenges including dietary adherence, pharmacological efficacy and safety, and individual gut microbiota variability to validate effective reduction techniques.

In conclusion, PE remains a severe condition with high maternal and fetal morbidity and mortality. Present-day management involves rapid birth delivery and aspirin functions as the primary preventive choice. The developing research regarding the role of gut microbiota-derived metabolites like TMAO opens up the possibility for enhancing diagnosis as well as treatment for PE. TMAO has been implicated in the pathogenesis of PE through multiple factors, including endothelial dysfunction, heightened inflammation, oxidative stress, and vascular abnormalities. Medical results for preeclamptic pregnant women could experience substantial improvement through correcting microbial imbalances and the modulation of TMAO levels. Medical practitioners need to examine and put into practice dietary modification programs together with probiotic treatment as well as pharmaceutical drugs in clinical settings to reduce TMAO-based damage, thus ensuring better maternal and fetal health.

As per the authors’ opinion, though the current treatments for PE are vital, we must expand our approach to include new biomarkers and targeted therapies like TMAO. Integration of gut microbiome research into clinical practice can revolutionize the way we treat pregnancy-related hypertensive disorders. It will lead not only to better treatment outcomes but also to more individualized care for high-risk pregnancies, which warrants the need for further trials to confirm TMAO’s clinical relevance and investigate therapeutic strategies to target it.

## Data Availability

Data sharing is not applicable to this article as no new data were created or analyzed in this study.

## Research registration unique identifying number (UIN)

The editorial didn’t have any human participants.
